# Snakebite Associated Thrombotic Microangiopathy and Recommendations for Clinical Practice

**DOI:** 10.3390/toxins14010057

**Published:** 2022-01-14

**Authors:** Tina Noutsos, Bart J. Currie, Eranga S. Wijewickrama, Geoffrey K. Isbister

**Affiliations:** 1 Global and Tropical Health Division, Menzies School of Health Research, Charles Darwin University, Darwin 0810, Australia; bart.currie@menzies.edu.au; 2 Division of Medicine, Royal Darwin Hospital, Darwin 0810, Australia; 3 National Hospital of Sri Lanka, University Medical Unit, Colombo 008000, Sri Lanka; erangasw@gmail.com; 4 Department of Clinical Medicine, Faculty of Medicine, University of Colombo, Colombo 008000, Sri Lanka; 5 Clinical Toxicology Research Group, University of Newcastle, Newcastle 2308, Australia; geoff.isbister@gmail.com

**Keywords:** snakes, snakebite, venom, thrombotic microangiopathies, acute kidney injury, hemolysis, schistocytes, neglected tropical diseases

## Abstract

Snakebite is a significant and under-resourced global public health issue. Snake venoms cause a variety of potentially fatal clinical toxin syndromes, including venom-induced consumption coagulopathy (VICC) which is associated with major haemorrhage. A subset of patients with VICC develop a thrombotic microangiopathy (TMA). This article reviews recent evidence regarding snakebite-associated TMA and its epidemiology, diagnosis, outcomes, and effectiveness of interventions including antivenom and therapeutic plasma-exchange. Snakebite-associated TMA presents with microangiopathic haemolytic anaemia (evidenced by schistocytes on the blood film), thrombocytopenia in almost all cases, and a spectrum of acute kidney injury (AKI). A proportion of patients require dialysis, most survive and achieve dialysis free survival. There is no evidence that antivenom prevents TMA specifically, but early antivenom remains the mainstay of treatment for snake envenoming. There is no evidence for therapeutic plasma-exchange being effective. We propose diagnostic criteria for snakebite-associated TMA as anaemia with >1.0% schistocytes on blood film examination, together with absolute thrombocytopenia (<150 × 10^9^/L) or a relative decrease in platelet count of >25% from baseline. Patients are at risk of long-term chronic kidney disease and long term follow up is recommended.

## 1. Introduction

Snakebite is a significant global public health issue. Globally there are an estimated 2.7 million cases of snake envenoming, with an estimated 81,000–138,000 deaths per year attributed to snakebite [1]. Snakebite is a condition which most affects low and lower-middle income countries, and people living below the World Bank’s international extreme poverty line [1,2]. The economic cost of snakebite is insurmountable in the countries most affected [1]. Snakebite victims are typically from tropical and sub-tropical rural and remote areas and subsistence farming populations. The World Health Organization (WHO) reclassified snakebite as a neglected tropical disease in 2017, with a target 50% death and disability reduction by 2025 [1].

Snake venoms cause a variety of potentially fatal clinical toxin syndromes affecting the nervous system (neurotoxicity), musculoskeletal system (myotoxicity), cardiovascular (cardiotoxicity), and blood clotting systems (haemotoxicity) [3]. Haemotoxicity and bleeding are important contributors to the morbidity and mortality of snakebite [4]. Coagulopathy is associated with numerous groups of snakes around the world and is triggered by a diverse variety of toxins. [5,6]. The main targets of snake venom haemotoxins are circulating blood clotting factors. Haemotoxic venoms can broadly be classified as procoagulant or anticoagulant in their mechanism of action [7]. The typical procoagulant coagulopathy of snakebite is a venom-induced consumption coagulopathy (VICC). It occurs due to activation of the clotting cascade at different clotting factor points specific to the venom toxins of the envenoming snake species. The major complication of VICC is haemorrhage, which can be fatal [5].

In a subset of patients with VICC, features of a thrombotic microangiopathy (TMA) develop. TMAs are a broad range of disorders defined clinico-pathologically by microangiopathic haemolytic anaemia (MAHA), evidenced by red blood cell fragments (schistocytes) on the peripheral blood film, and thrombocytopenia [8]. Histologically the TMAs are marked by small vessel wall damage and micro-thrombosis, and resultant vaso-occlusive ischaemic end organ injury [9]. Snakebite-associated TMA has been described in a variety of snake species known to cause VICC across all inhabited continents of the world [10,11,12,13,14]. Snakebite-associated TMA appears to be specifically associated with predominant acute kidney injury (AKI) [10,15,16,17,18], in contrast to the major complication of VICC, i.e., bleeding [7].

Snakebite-associated TMA has been variably compared to other TMAs such as thrombotic thrombocytopenic purpura (TTP) and haemolytic uraemic syndrome (HUS) [18,19]. Snakebite-associated TMA resembles HUS with its predilection for renal end-organ damage, but any association beyond this common presenting feature has not been established. Some studies have reported the AKI of snakebite associated TMA as self-limiting [17,20], whilst other small studies have claimed effectiveness of intervention with therapeutic plasma exchange (TPE) [12,21,22,23]. TPE has a strong evidence base for preventing the high mortality in TTP [9]. However, its use in other TMAs is often based on limited to no evidence of benefit [24]. TPE is resource intense, requires large volumes of blood donation (plasma) products and carries some risk of adverse reactions [25,26]. Any correlation between snakebite associated TMA and TTP with respect to aetiology or treatment is unproven.

Better elucidating the features of snakebite associated TMA and best practice with respect to treatment is of global importance [3]. Historically, snakebite associated TMA has been mostly described in single case reports or small case series [12,17,27]. Until recently, little was known about its natural history, outcomes, pathophysiology, or best treatment [11,17,18]. More recently a small number of retrospective and prospective cohort studies on snakebite associated TMA from Sri Lanka, India and Australia have been published [15,28,29]. An improved understanding of snakebite associated TMA and best treatment approaches will allow the best allocation of resources and interventions, particularly in the parts of the world most affected by snakebite: resource limited settings [4].

Here, we review the epidemiology, presenting clinical and laboratory features, outcomes, and role of interventions including antivenom and TPE, and provide clinical practice recommendations based on latest evidence.

## 2. Epidemiology

Snakebite associated TMA is reported globally, in a wide variety of envenoming snake types, within a subset of patients envenomed by snakes associated with procoagulant haemo-toxic venoms causing VICC [30]. Although snakebite-associated TMA has previously been thought rare, it in fact occurs in a significant proportion of patients with VICC and in a diverse range of haemo-toxic snake genera/species across all populated continents of the world. Depending on the case definition of TMA applied, it has been reported in 19% of snakebites admitted to hospitals in India [31], 5 to 10% of *Hypnale* bites [16,32], and 9% of envenoming cases from Australian snakes, including 17% of Australian brown snake envenomings, 26% of all Australian taipan envenomings, and 8% of all Australian tiger snake envenomings [29]. Worldwide, most cases have been reported from India, Sri Lanka and Australia, with smaller reports from many other countries globally [30]. It is highly likely that the condition is significantly under-reported in many parts of the world. TMA is more commonly reported in males compared to females, which reflects snakebite being more common in males more broadly, and there is no evidence that male sex is a significant risk factor for the development of TMA. Patients of all ages are affected, although a recently published Australian Snakebite Project prospective cohort study suggests that increasing age may be a risk factor for the development of TMA, with TMA patients significantly older than non-TMA patients with VICC alone [29].

## 3. Association with Envenoming by Snake Genera/Species Causing VICC

Snakebite associated TMA appears to be associated with VICC, irrespective of the snake or snake venom. VICC is characterised by procoagulant toxins activating various clotting factors, leading to activation of the coagulation cascade, factor consumption and a resultant consumption coagulopathy [4,7]. VICC presents with a reduced or unrecordable fibrinogen; prolonged or unrecordable PT (or its international normalised standardised equivalent, the international normalised ratio (INR) [33]) and activated partial thromboplastin time (APTT); and an elevated D-dimer [34]. It is an acute and transient coagulopathy associated with a risk of bleeding, worse in snake venoms that also contain metalloproteinases. Recovery from VICC occurs at a rate consistent with clotting factor resynthesis after inactivation or neutralisation of the venom toxins [34]. VICC is consistently present in available studies of snakebite-associated TMA, in which reported serial coagulation testing allows classification of VICC, and typically precedes the development of TMA features (MAHA, thrombocytopenia and AKI) [29,30,31,35,36,37]. In Australian snakebite, all reported TMA cases have occurred in patients with preceding VICC, either complete or partial [29].

In studies with more heterogeneously reported clotting tests, it is difficult to definitively categorise the coagulopathy preceding TMA as definite VICC, but the coagulopathy is nevertheless most consistent with VICC [28,38]. Smaller studies of snakebite associated TMA in resource limited settings report the 20-min whole blood clotting time (WBCT20) rather than tests enabling classification of VICC [39]. Although, the WBCT20 is endorsed by the WHO, studies have demonstrated that it is less accurate and reliable than formal laboratory measures of clotting, such as the INR/PT, APTT and fibrinogen [40]. These limitations affect WBCT20 sensitivity and specificity in diagnosis of coagulopathy after snakebite [41]. However, WBCT20 remains in widespread use globally where laboratory coagulation analyser testing is not readily accessible. Acknowledging these limitations of the WBCT20, in resource limited settings in which formal laboratory testing for INR/PT, APTT and/or fibrinogen are not available, patients with suspected snakebite and a prolonged WBCT20 should also be considered at risk for developing TMA.

**Figure 1:**
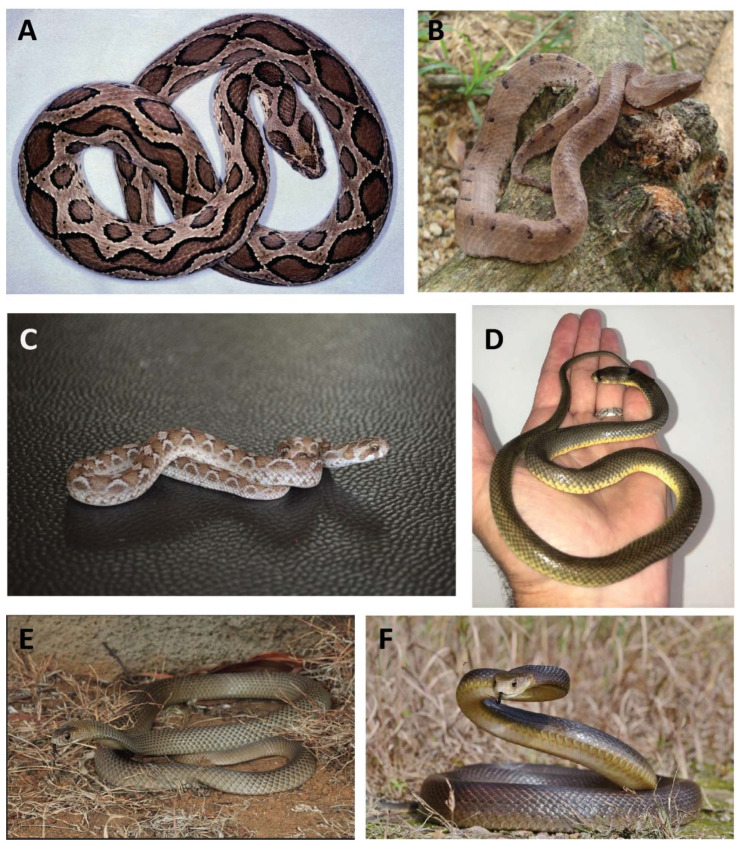
Snake species associated with thrombotic microangiopathy: (**A**,**B**) From Asia, (**A**) Russell’s viper (*Daboia russelii*); and (**B**) Hump nosed viper (*Hypnale hypnale*); (**C**) from Asia and Middle East, saw scaled viper (*Echis carinatus*); from Australia (**D**–**F**) Northern or tropical northern brown snake (*Pseudonaja nuchalis*); (**E**) Eastern or common brown snake (*Pseudonaja textilis*), and (**F**) coastal taipan (*Oxyuranus scutellatus*).

Snake species causing TMA are those with a venom composition known to cause VICC, predominantly vipers and certain Australasian elapids (Table 1) (Figure 1) [4]. The haemotoxins associated with VICC and snakebite associated TMA include a variety of clotting factor activators: prothrombin activators (*Echis* spp., *Pseudonaja* spp., *Oxyuranus* spp., *Notechis scutatus*, and *Hoplocephalus* spp.); factor V/factor X activators (*Daboia* spp.); thrombin like enzymes +/− other mechanisms; (*Hypnale* spp., *Cerastes cerastes*, *Bitis arietans*) and thrombin like enzymes/prothrombin/factor X activators (*Bothrops jararaca*) [7,42,43]. Of note, definite cases of TMA have not been reported in every snake species causing VICC (e.g., *Calloselasma rhodostoma*, *Trimeresurus* spp. Table 1) [30]. Given the likelihood that TMA is significantly under-reported in many parts of the world, this lack of reported cases does not completely exclude an association with these species.

TMA does not occur in snakes causing anticoagulant coagulopathy, including black snakes (*Pseudechis* spp.) from Australia [29,44]. These are associated with a raised APTT with or without a mild elevation in the INR, and a normal D-dimer [45]. TMA has not been reported in these snakebites (Table 1) [29].

## 4. Snakebite Associated TMA Is Defined by MAHA and Thrombocytopenia

**Figure 2:**
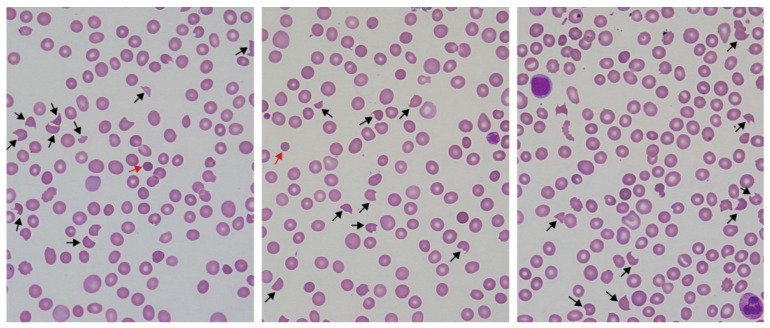
Peripheral blood films from Australian elapid envenomings with thrombotic microangiopathy. Microangiopathic haemolytic anaemia is defined by the presence of blood film schistocytes (black arrows). Red cell morphological changes in microangiopathic haemolytic anaemia may also include micro-spherocytes (red arrows), which are secondarily derived from schistocytes, as defined by the International Council for Standardization in Haematology [53,54]. Derived from Noutsos et al. [55].

Snakebite-associated TMA typically presents with a MAHA evidenced by anaemia with >1.0% red cell fragments (schistocytes) on the peripheral blood film, together with thrombocytopenia (Figure 2) [28,29,44]. Anaemia, raised haemolysis markers (lactate dehydrogenase [LDH], and thrombocytopenia are almost universally present, although many studies have used differing TMA case definition, including a case definition of TMA as MAHA alone [29,30], MAHA plus thrombocytopenia [28], and MAHA plus thrombocytopenia and AKI [15], so selection biases between studies due to heterogeneity of study case definitions limits comparisons between them. The thrombocytopenia and anaemia are usually moderate, with the platelet nadir typically between 20 and 100 × 10^9^/L, and both typically reach a nadir between 2 to 7 days post-bite [28,29,30]. Importantly, a mild and transient thrombocytopenia may occur in snakebite more broadly [52], underscoring the importance of correlation with the presence of MAHA, which is the *conditio sine qua non* for the diagnosis of TMA specifically.

Using data from the Australian Snakebite Project, when we applied a definition of snakebite associated TMA as MAHA (anaemia with blood film schisto-cytosis >1.0% red cell fragments), 90% of cases also had thrombocytopenia (platelets < 150 × 10^9^/L), with a small subset (10%) of outliers with a normal platelet count nadir. Of this small subset, 8% had a relative (>25%) drop in their baseline platelet count and the remaining 2% had neither [29]. Similarly, in a two-year prospective cohort study of patients with snakebite and severe AKI admitted to a Sri Lankan tertiary care hospital, of 59 patients with snakebite and AKI, 45 had TMA defined by MAHA, thrombocytopenia and AKI, 11 had AKI and isolated thrombocytopenia without MAHA, and two had AKI with MAHA without thrombocytopenia [15]. These findings support the hypothesis that snakebite-associated TMA is a spectrum disorder with respect to presenting laboratory features.

## 5. Peripheral Blood Film Examination for Schistocyte Quantitation as a Diagnostic Tool for Snakebite-Associated TMA

**Figure 3:**
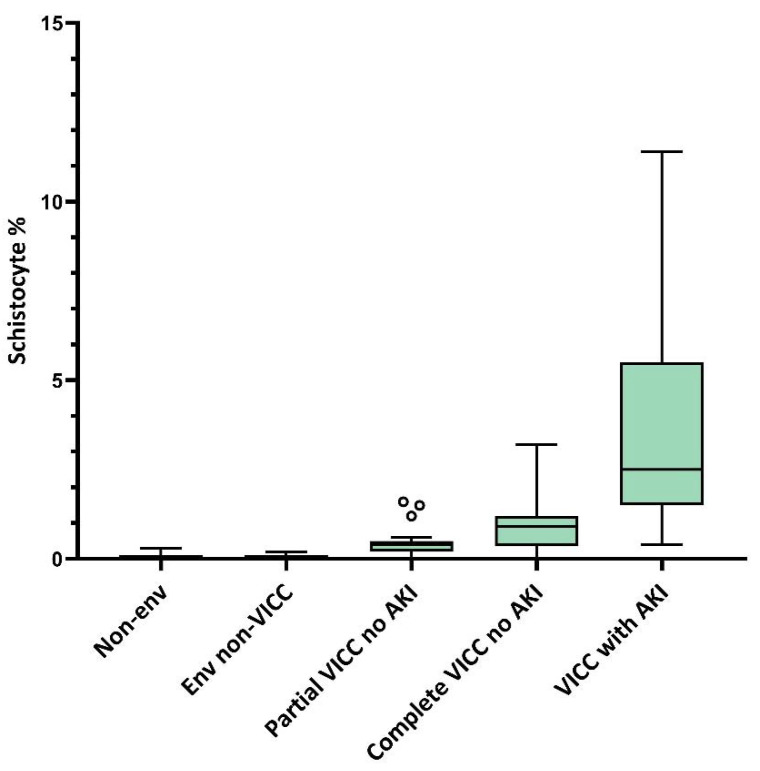
Peak blood film schistocyte percentage in Australian elapid envenomings by clinical toxin syndrome. Tukey plot of medians and interquartile ranges (boxes), 5% and 95% centiles (whiskers), and outliers (ο). Kendall’s tau b correlation coefficient 0.48, *p* < 0.001. Env: envenomed; VICC: Venom-induced consumption coagulopathy; AKI: Acute kidney injury. Modified from Noutsos et al. [44].

Examination of the peripheral blood film for manual quantitation of schistocytes has diagnostic utility for TMA diagnosis in suspected snakebite. Two studies from Australia and India provide evidence supporting a role for schistocyte quantitation in snakebite [28,44,55]. This is in addition to several studies on the diagnostic utility of schistocyte quantitation for the diagnosis of TMA more broadly in non-snakebite settings [54,56,57,58,59]. Schistocytosis in snakebite is significantly predictive of the development of AKI in Australian snakebite presenting with VICC, with AKI shown in multiple studies to be the major complication of snakebite-associated TMA [44]. Schistocyte quantitation in Australian elapid envenomings is significantly correlated with the ordered groups of non-envenomed, envenomed non-VICC, partial VICC without AKI, complete VICC without AKI, and VICC with AKI cases (Figure 3). A 1.0% schistocyte cut-off is 90% sensitive and 71% specific for diagnosing AKI in patients with VICC in Australian snakebite. Schistocytes on blood films are detectable and at ≥ 1.0% by 24 h in Australian elapid envenomings, offering an early diagnostic tool for TMA diagnosis [44]. One retrospective cohort study from India of 103 snakebites admitted with AKI found that TMA defined by the presence of >1.0% blood film schistocytes and thrombocytopenia was associated with more severe AKI in *Hypnale* spp. envenoming compared to snakebite cases admitted with AKI and no TMA [28].

Findings on intra- and inter-observer agreement for blood film manual schistocyte quantitation show it is moderately reliable and reproducible, underscoring the importance of repeated blood film monitoring in the setting of suspected TMA in snakebite. Studies have shown that manual blood film schistocyte quantitation in Australian snakebite shows moderate to high reliability between different examining microscopists, and substantial to near perfect intra-observer agreement on repeated examinations by the same microscopist [55,60,61,62,63]. Microscopists who are calibrated and trained can perform the procedure with reasonable inter- and intra-observer agreement. Cases in which there is a clinical suspicion of TMA should undergo serial blood film examinations over the first 24 h [54].

In the regions of the world where snakebite is most prevalent, access and/or resourcing of hospital diagnostic laboratories are more likely to be limited with respect to laboratory equipment and reagents, well developed local guidelines, quality control and laboratory scientist training, making schistocyte quantitation more difficult. Nevertheless, in the broader global health context, manual blood film microscopy and careful examination of erythrocytes remains an important test, for example in the diagnosis and speciation of malaria. The WHO includes peripheral blood film examination, and Wright, May-Grünwald or Giemsa staining for blood films in its model list of essential in vitro diagnostics for primary health care where a good quality microscope is available, and for all district, regional, provincial and specialised hospitals or laboratories [64]. The International Council for Standardization in Haematology have a published publicly available international consensus guideline on manual blood film schistocyte quantitation from 2012, which was recently updated in 2021 [53,54]. In addition, the WHO produces multiple written guidelines and illustrated bench aids with respect to manual blood film methods and the identification of abnormal red cell morphology [65,66].

## 6. Acute Kidney Injury Is the Predominant End Organ Damage in Snakebite-Associated TMA

Studies consistently support AKI being the predominant end-organ site of injury in snakebite-associated TMA, but estimating the true prevalence and spectrum of AKI in snakebite-associated TMA is challenging [30]. Many studies have significant selection bias and heterogenous case definitions of TMA, some of which include AKI as part of the case definition. AKI in snakebite more generally has also been previously attributed to other causes including direct venom toxicity and consequent to shock. The historical literature has been heterogenous with respect to snakebite with AKI definitions and terminology, with renal outcomes often poorly defined and reported. In addition, few existing studies have a design which clearly represents the whole experience of research investigators, with many case series or case reports only. Those with a cohort design and clear case definitions have often been based in renal tertiary referral hospitals and carry the risk of selection bias.

A recent systematic review found AKI in 94% of snakebite-associated TMA cases after the exclusion of case reports [30], with other non-renal end organ injury being very uncommon. AKI was reported in 77% of snakebite-associated TMA cases from one single centre retrospective cohort study from India [31], and in 94% of TMA cases in the largest prospective cohort study of snakebite-associated TMA from Australia when applying a definition of TMA as MAHA with schistocytes on the peripheral blood film [29]. Other (non-renal) end-organ injury (for example heart, brain, pituitary, lung) in snakebite-associated TMA has been reported but is rare [67]. When reported it is often not clearly attributable to TMA specifically, and/or occurs in the presence of other potential causes such as hypotension, cardiovascular collapse, or cardiac arrest [29,30].

The AKI in snakebite-associated TMA commonly requires dialysis support in the acute setting. Three studies from India which report AKI stage and dialysis requirement with a low risk of selection bias found dialysis-requiring AKI in 69%, 86%, and 95% of snakebite associated TMA with AKI [28,31,35]. This compares to dialysis-requiring AKI in 34% of Australian snakebites with TMA and AKI in a prospective cohort study, which comprehensively enrolled suspected and confirmed snakebites presenting to over 200 centres across the country. This Australian study found a much greater spectrum of AKI severity including cases with only mild and moderate AKI [29]. The incidence of dialysis support requirement for snakebite-associated TMA is likely to be over-estimated in many existing studies owing to study designs, in which patient recruitment occurs from renal referral centres, with some studies including AKI in their case definition, or in resource limited settings where the likelihood is that milder forms of AKI are missed due to lack of testing for the traditional markers of AKI (creatinine testing and urine output monitoring).

Outside of snakebite specifically, AKI is a common and serious complication of inpatient hospitalisations [68]. AKI affects 20% of hospital patients, and up to 50% of ICU patients [69]. Renal injury is often a silent disorder, particularly in its early stages [70]. With increasing severity, symptoms and signs related to volume overload (including peripheral oedema, pulmonary oedema and raised central venous pressure); proteinuria and consequent hypo-albuminaemia and loss of blood oncotic pressure; metabolic acidosis; hypo-natraemia; hyper-kalaemia; hyper-phosphataemia; hypo-calcaemia; uraemia (which can cause encephalopathy, pruritis, pericarditis, and bleeding due to platelet dysfunction); drug toxicity; and ultimately death, can develop [71]. The prognosis for AKI is related to multiple factors, including the stage of AKI, the underlying aetiology of the AKI, the duration of the AKI, concurrent renal insults such as intravascular volume depletion, haemo-dynamic instability, concomitant sepsis, inflammation or exposure to nephrotoxic drugs, patient age, and comorbidities such as antecedent chronic kidney disease, diabetes, and hypertension [72].

AKI is traditionally defined by a sudden rise in serum creatinine and reduction in urine output over hours to days, though there is emerging evidence for new biomarkers in the earlier detection of AKI, including in snakebite [73]. These new biomarkers may allow earlier and more specific identification of AKI. In snakebite specifically, recent studies have shown three new biomarkers (serum cystatin C, urine neutrophil gelatinase-associated lipocalin, and urine clusterin) are superior to creatinine in the identification of AKI in snakebite in the 4–8 h post-bite [74,75]. Whether new biomarkers of AKI may serve as an early diagnostic tool for the AKI of snakebite-associated TMA is unknown.

Management of AKI of any aetiology involves the identification and treatment of its underlying cause; judicious avoidance of further renal insults; the prevention and expedient treatment of complications including hyper-kalaemia, acid base disturbances, and fluid overload; and intense supportive care including nutritional support, and dialysis when needed [70,71]. Management of AKI in the setting of snakebite should be carried out along the same lines, irrespective of whether or not TMA is the cause. Maintenance of haemo-dynamic stability and prevention and correction of volume depletion are paramount [71]. Estimating the amount of fluid needed to administer can be challenging, especially in the setting of AKI associated with oliguria or anuria, where excess fluid administration can easily lead to fluid overload. Wherever possible, nephrotoxic medicines or other substances (for example iodine contrast commonly used in computed tomography scans, non-steroidal anti-inflammatory drugs, gentamicin, vancomycin, and other nephrotoxic antibiotics) should be avoided, and where appropriate therapeutic drug levels and doses carefully monitored and adjusted [73]. For severe AKI, patients may require dialysis [71]. Peritoneal dialysis, in comparison to haemo-dialysis, has clearance limitations, challenges with fluid removal, and complications such as peritonitis [70]. It is rarely used for AKI patients in well-resourced health care settings but is more commonly used for patients with AKI in resource limited settings, including for AKI in the global settings where snakebite is most common [76,77,78].

Blood film studies in Australian elapid bites support TMA as the predominant cause of AKI in snakebite, although studies of *Hypnale* spp. envenoming show that AKI in association with snakebite may also be due to other (non-TMA) causes. Studies from Australia also show that AKI in envenomed non VICC cases is exceedingly uncommon, making TMA the most likely aetiology of AKI in snakebite, particularly for envenoming by Australian elapids, where severe myotoxicity with rhabdomyolysis is relatively rare [14]. However, this may differ in an international setting of snake species more associated with myotoxicity and secondary rhabdomyolysis. Studies from Sri Lanka report significant subsets of patients with AKI post *Hypnale* spp. envenoming without TMA (48–70%) suggesting a more diverse range of causes for AKI than TMA alone [16,32].

## 7. In Hospital Outcomes: Overall Survival and Dialysis Free Survival

The largest studies of hospital outcomes in snakebite-associated TMA from Sri Lanka, India and Australia show that most TMA patients survive, with overall survival between 93–100% in the three largest studies from India and Sri Lanka [15,28,31], and 96% in the Australian Snakebite Project [29]. This compares to 44% overall survival in a Burmese study which reported cases from a highly selected cohort of patients proceeding to renal biopsy or autopsy [79]. Deaths are infrequent, usually attributable to major haemorrhage, and/or cardiogenic shock or cardiac arrest and/or associated secondary multiorgan failure.

Most cases of snakebite-associated TMA with AKI requiring dialysis support recover and subsequently achieve dialysis free survival (DFS). DFS reported in Indian and Sri Lankan studies is between 80–95% [15,28,35], and in Australia 97% of snakebite-associated TMA cases initially requiring dialysis achieved DFS [29].

Patients often require early and high level intensive supportive care. In the Australian Snakebite Project cohort, this included basic and advanced life support including but not limited to emergency resuscitation and management of haemo-dynamic instability, intensive care, inotropes, dialysis, and (often in the setting of concurrent neurotoxicity and bulbar paralysis), intubation and ventilation. Small case reports and case studies from low-middle income countries and/or resource-limited settings report similar findings with respect to clinical presentations, which would require similar level intensive care support, although resourcing limitations do not always allow for their provision.

## 8. Role of Interventions: Antivenom and TPE

Interpretation of data is limited regarding evidence for or against the administration and timing of antivenom in preventing the development of TMA in patients with VICC. There is no clear evidence for antivenom preventing the development of TMA in patients presenting with VICC, although existing data are both limited and/or confounded, including by treatment bias and inter-related variables such as time to hospitalisation and access to best supportive care. TMA has been reported in envenomings by snake species where effective antivenom is not available (e.g., *Hypnale* spp.), but is also found in bites where antivenom is available and effective if given early (e.g., *D. russelii*, Australian elapids) [29,31,32]. Similarly, for cases that develop TMA, there is no clear evidence that antivenom administration carries benefits with respect to the outcomes of AKI or overall survival [29,30,31,32].

This paucity of clear evidence supporting a role for antivenom in snakebite-associated TMA is consistent with the lack of quality evidence for antivenom in the treatment of VICC itself [42,80,81,82]. Quality control assays for research/licensing of new antivenoms and manufacturing for new batches of antivenom typically assess the neutralising efficacy of antivenoms via both in vitro and in vivo animal studies. In such studies, venom and differing doses of antivenom are typically pre-incubated together prior to injection and assessment of neutralisation of venom-specific effects such as death or haemorrhage [83,84]. However, in real world settings, it is likely that administration of antivenom to patients in order to prevent haemotoxic effects of snakebite is so time critical that it is administered too late after envenoming to prevent either VICC or TMA. However, studies of VICC in *Echis* spp. bites specifically have demonstrated that antivenom does improve the recovery of VICC and so may potentially reduce the incidence of TMA [85]. Despite these uncertainties, antivenom remains the standard of care in snakebite envenoming [3,84,86]. The limitations with respect to preventing TMA do not detract from the central role of early intervention with antivenom at the first sign of envenoming, which is an essential component of emergency treatment.

Globally, there is no evidence of benefit with TPE treatment in snakebite-associated TMA in dialysis requiring AKI cases with respect to either overall survival, time to dialysis independence, or long-term dialysis free survival. While recommendations with respect to TPE are limited by the quality of available studies, both prospective and retrospective cohort studies from Sri Lanka, India and Australia have failed to show a benefit from TPE [15,28,29,30]. A limitation of these studies is insufficient power to rule out a type 2 error. Nonetheless they provide the most robust evidence to date on the question of the role of TPE in snakebite-associated TMA. For snakebite-associated TMA cases with AKI requiring dialysis support, this dialysis requirement typically continues for days to weeks, after which time it resolves. While the evidence needs to be interpreted with caution given low sample sizes, TPE cannot be recommended as routine medical practice for snakebite-associated TMA. TPE is resource intense, requires specialised blood donor products (plasma), carries some risk including of allergic reactions, and is expensive. These issues are particularly important to consider in the context of the regions of the world where snakebite is most common: resource limited settings where health care expenditure is best directed towards evidence-based practice.

This recommendation against the routine use of TPE in snakebite-associated TMA is in keeping with existing evidence and best practice for the management of the other non-TTP and non-HUS causes of TMAs, such as those due to toxins and medications. TMA is associated with a variety of drugs, toxins, and medical conditions. Notable examples are TMA associated with malignancy, haemato-poietic stem cell transplant, renal allografts, and medications including cyclosporine A and tacrolimus [8]. In these cases, the preferred nomenclature is “secondary TMA” [8,87]. The means by which many of these conditions lead to TMA is generally less well characterised. In these secondary TMAs, ADAMTS-13 activity is generally not reduced, and inhibitor testing is typically negative. Proposed aetiologies for these other TMAs include direct endothelial injury [24,88]. Management of secondary TMA generally entails treatment or removal of the precipitating cause, where possible. [24,87]. With rare exception, there is no established role for TPE in these secondary TMAs. TPE has been shown in most studies to be either of uncertain benefit, or ineffective [24]. Ticlopidine associated TMA, associated with autoantibodies to ADAMTS-13, is one possible exception to this rule [89].

## 9. Long-Term Survival and Risk of CKD

Globally most studies in snakebite-associated TMA and AKI, or all cause AKI in snakebite, have had incomplete or limited follow up-data with respect to long-term renal outcomes. Existing studies also share the limitations associated with the literature in snakebite VICC and snakebite-associated TMA: they are mostly small, retrospective, and use inconsistent and often ill-defined nomenclature regarding renal dysfunction and case definitions of AKI [30].

Despite the favourable outcomes with respect to overall survival and DFS in snakebite-associated TMA, there is emerging evidence that patients are at significant risk of chronic kidney disease (CKD). In the Australian Snakebite Project, over half of patients with AKI requiring initial dialysis support for whom there was long-term follow-up had ≥ Stage 3 CKD, even though they initially achieved DFS [29]. Further indirect evidence of a risk of CKD in snakebite-associated TMA comes from a recent prospective study from India of 785 snakebite cases. While not explicitly reporting on snakebite-associated TMA, in this study mortality was 21.5% in cases with haemo-toxicity and AKI, and relative risk of death in KDIGO stage 3 AKI was 4.45 (95% CI 1.14–17.42) compared to the cases with haemo-toxicity but no AKI. Follow up for >3 months was available for 73 patients, of which one third of patients with AKI developed long term complications including CKD [90]. These findings are not surprising, given the strong epidemiological link between AKI of all causes and the later development of CKD [70]. CKD is a complex disease associated with significant morbidity and mortality, and long-term follow-up of snakebite-associated TMA patients is therefore critical. In advanced CKD, in addition to the loss of essential kidney functions involving fluid volume, electrolyte and acid base balance, loss of the renal endocrine function also develops, marked by renal anaemia and secondary hyperparathyroidism [70,91,92]. Patients with CKD have a significantly increased incidence of cardiovascular disease, and a much reduced life expectancy [92]. These issues underscore the importance of long-term follow up of snakebite associated TMA patients, for the purposes of CKD secondary and tertiary prevention, and chronic disease management as required.

## 10. The Aetiology of Snakebite Associated TMA Remains Unclear, but It Is Not DIC, TTP, or HUS

The aetiology of snakebite associated TMA remains unclear. While snakebite-associated TMA has some presenting features in common with HUS, it is distinctly different from both HUS and TTP. Few cases of snakebite associated TMA have had ADAMTS-13 testing, but it has been normal when tested. It is unlikely that ADAMTS-13 deficiency plays a role in snakebite associated TMA, although given the small numbers tested the possibility of a role for ADAMTS-13 cannot be excluded. Most other secondary causes of TMA due to drugs and toxins are not associated with ADAMTS-13 deficiency, with rare exceptions including ticlopidine which induces ADAMTS-13 antibodies [30,89]. Similarly, complement is also rarely tested, and while normal in the snakebite-associated TMA cases reported, there are too few cases tested to draw any conclusions. Additionally, complement testing in isolation is insufficient to rule out a complement mediated process. In atypical HUS (aHUS), complement testing such as C3 and C4 may be normal, and mutational analysis of complement regulated genes is required together with clinicopathological correlation for its diagnosis [93]. Further studies are needed to fully elucidate the cause of snakebite associated TMA.

Some studies to date continue to refer to snakebite-associated TMA as either TTP or HUS [32,94]. With respect to internationally accepted nomenclature, TMA is a pathological term used broadly to describe a range of conditions with differing aetiologies, presenting either clinically with MAHA and thrombocytopenia, or defined pathologically by the microvascular vessel wall damage and micro-thrombosis [8]. TTP and HUS are specific types of TMA which clinically present with a predominance of neurological and renal end-organ injury respectively, but they are specifically defined further: TTP is specifically caused and defined by deficient ADAMTS-13 activity testing (<10%), and HUS as either infection associated or complement mediated, and defined by testing for the infectious cause (or shiga-like toxin) by stool culture/PCR, or by complement mutational analysis. While a negative complement mutational analysis does not exclude complement mediated HUS, diagnosis then involves excluding all other precipitating secondary causes of TMA [8], such as malignancy, transplant, pregnancy, and other drugs/toxins. It is more appropriate that snakebite-associated TMA is considered a secondary TMA, not as HUS. This recommendation is consistent with current consensus statements on the standardisation of terminology used in TMA [8,87].

While the features of both VICC and snakebite associated TMA fit the traditional diagnostic scoring criteria of disseminated intravascular coagulation (DIC), VICC and snakebite-associated TMA are distinct from DIC, with different aetiology, pathophysiology, clinical presentation and prognosis [95,96]. DIC is a common coagulopathic state seen in conditions such as sepsis, malignancy, burns, polytrauma and obstetric emergencies such as amniotic fluid embolism and placental abruption [96]. It is defined by the presence of thrombocytopenia, prolonged prothrombin time (PT), reduced fibrinogen and elevated fibrin-related markers such as fibrin degradation products or D-dimer. Central to its pathophysiology is a consumptive coagulopathy triggered by tissue factor mediated activation of factor VII pathways and thrombin generation [96]. Coagulopathy, as evidenced by prolonged PT and/or APTT, is characteristically present during DIC, but in snakebite the prolonged PT and/or APTT of VICC typically normalise prior to the overt development of TMA. While the aetiology of snakebite-associated TMA remains unknown, its triggering exposure (procoagulant snakebite venom toxins/enzymes) is distinct from the factor VIIa/tissue factor pathway aetiology of DIC. Blood film schisto-cytosis ≥1.0% is sensitive and specific for snakebite-associated TMA, and this is distinct from studies on schistocyte quantification in DIC, where schistocytes are present but to a mild degree only and typically less than 1.0% [62]. In contrast to VICC and snakebite-associated TMA, multi-organ failure owing to systemic vessel micro-thrombosis in end organs is a major feature of DIC [95,96]. Autopsy examination in cases with multi-organ dysfunction or failure due to DIC show intravascular thrombi and fibrin deposition, organ ischaemia and necrosis in addition to diffuse haemorrhage [96]. This is responsible for the significant morbidity and mortality seen in DIC [97]. Overall survival and DFS are much more favourable in snakebite-associated TMA when compared with DIC.

## 11. Conclusions and Recommendations for Practice

Based on all available evidence internationally, we make the following recommendations with respect to snakebite-associated TMA:

1. Snakebite-associated TMA should not be included within inclusion criteria for DIC diagnostic scoring systems;
2. Scoring systems such as those used for the diagnosis of DIC (by the International Society on Thrombosis and Haemostasis) [98], and the PLASMIC score used for predicting ADAMTS-13 < 10% in suspected TTP [99], should not be applied in snakebite envenoming cases;
3. The terms TTP and HUS should not be used in association with snakebite. Snakebite should be considered as a condition associated with secondary TMA, but distinct from both TTP and HUS. The preferred term is snakebite-associated TMA, or alternatively, TMA secondary to snakebite;
4. Where resources allow, a manual blood film for quantitation of schistocytes is recommended in all patients presenting with snakebite envenoming and VICC from haemo-toxic snakebites. It is recognised that this will not always be possible in the global settings where snakebite is a significant public health issue. In this instance serial creatinine testing, and/or careful observation of urine output are important for the detection of AKI, the main end organ injury of snakebite associated TMA;
5. A definition of snakebite-associated TMA consisting of MAHA with ≥1.0% schistocytes, together with either thrombocytopenia (platelets < 150 × 10^9^/L) or a >25% decrease in platelets from baseline, is recommended. When applying this definition, it must be recognised that this is a spectrum disorder and that there is a very small subset of outliers at risk of AKI who will not meet all features of this definition;
6. While evidence for a role of antivenom in the prevention of TMA in patients presenting with VICC is unclear, early intervention with antivenom at the first sign of envenoming remains the standard of care in snakebite;
7. TPE for snakebite-associated TMA cannot be recommended for routine medical practice. This is consistent with most other types of toxin- or drug-associated TMA. Whilst the role for TPE in TTP is indisputable, there is generally either uncertain evidence for benefit, or evidence which demonstrates that TPE is either ineffective or harmful in most secondary causes of TMA not associated with ADAMTS-13 deficiency, such as snakebite [24];
8. Globally, resources for the care of patients with suspected or confirmed snakebite-associated TMA should be directed towards best supportive care. This is particularly critical in low resource settings including the rural and impoverished regions of the world where snakebite is most common. Supportive care includes antivenom for envenomed patients; judicious attention to measures which mitigate exposures to additional renal insults such as volume depletion, haemo-dynamic instability and nephrotoxic drugs; intensive care as required; and dialysis when needed and as local resources allow. Patients may require intensive care support for other clinical toxicities of snakebite such as neurotoxic paralysis, and stabilisation to avoid other contributors to renal injury including hypotension;
9. As with other causes of AKI, patients with TMA are at risk of subsequent CKD after initial recovery and long-term follow-up is strongly recommended. This should include monitoring of renal function, avoidance and management of other renal risk factors, and CKD management as required.

## Figures and Tables

**Table 1:** Snakes grouped by mechanism of coagulopathy and reported snakebite-associated TMA. Derived from Isbister et al., Maduwage et al., Berling et al. [5,7,46].

Coagulopathy Type	Toxin Mechanism of Action	Factor and Haematologic Effects	Snake Species	Common Name	Distribution	Definite TMA Reported *
VICC	Prothrombin activators	Fibrinogen, FII, FV, FVIII	Echis carinatus **	Saw-scaled viper	Asia/Middle East	Yes [47]
			Echis ocellatus **	West African carpet viper	Africa	Yes [38]
			*Pseudonaja* spp.	Brown snake	Australia	Yes [29]
			*Notechis scutatus*	Tiger snake	Australia	Yes [29]
			*Tropidechis carinatus*	Rough-scaled snake	Australia	Yes [29]
			*Hoplocephalus* spp.	Broad-headed snakes	Australia	Yes [29]
			*Oxyuranus scutellatus*	Coastal taipan	Australia	Yes [29]
	Factor X activator	Fibrinogen	*Vipera aspis*	European asp/asp viper	Europe	No [30]
	Factor V/Factor X activators	Fibrinogen, FV, FX	*Daboia russelii*	Russell’s viper	Asia	Yes [15]
	TLEs	Fibrinogen	*Hypnale* spp.	Hump nosed viper	Asia	Yes [16]
			*Atheris* spp.	Bush vipers	Africa	Yes [48]
			*Bitis arietans*	African puff adders	Africa	Yes [49]
			*Trimeresurus* & related genera	Green pit vipers & others	Asia	No [30]
			*Calloselasma rhodostoma*	Malayan pit viper	Asia	No [30]
			*Crotalus atrox*	Western diamondback rattlesnake	North America	No [30]
			*Cerastes vipera*	Sahara sand viper	Africa/Middle East	No [30]
	TLEs/Other	Fibrinogen, FV	*Cerastes cerastes*	Saharan horned viper	Africa/Middle East	Yes [50]
	TLE, PTA, factor X	Fibrinogen, FII, FV, FVIII	*Bothrops jararaca*	Jararaca	South America	Yes [51]
Anticoagulant type	Inhibitory action against FX, FII (prothrombin), and platelets	Elevated APTT	*Pseudechis australis*	Mulga	Australia	No [29]
			*Pseudechis porphyiacus*	Red-bellied black snake	Australia	No [29]
			*Pseudechis* spp.	Other black snakes	Australia	No [29]
	Protein C activator	Unknown	*Agkistrodon* spp.	Southern Copperhead	North America/Central America	No [30]

* Definite TMA defined by reporting of histological features of TMA and/or blood film schistocytes/red cell fragments. ** Echis species names and their respective distributions are under active discussion and revision. VICC: Venom induced consumption coagulopathy; TMA: Thrombotic microangiopathy; F: Factor; TLE: Thrombin-like enzymes; PTA: Prothrombin activator; APTT: Activated partial thromboplastin time.

## Data Availability

Not applicable.
